# 

**Published:** 1879-02

**Authors:** 


### CONTENTS:

Article I.—Inflammation, Abscess and Necrosis, their Cause and Treatment. By J. M. Porter, D. D. S. .... 433

Article II.—Treatment of Deciduous Teeth. By W. E. Royce, D. D. S. .... 446

Article III.—American Academy of Dental Science By E. N. Harris, D. D. S. .... 451

Article IV.—Dental Neuralgia. By G. V. Black, D. D. S. .... 456

Article V.—The Alcohol Question in England .... 461

Article VI.—The Care of the Eyes .... 464

Article VII.—Epithelioma—Blepharoplasty .... 466

Article VIII.—The Uses of Pain .... 467

Article IX.—College Commencements and Alumni Meetings—

Baltimore College of Dental Surgery .... 468

Alumni Meeting of Baltimore College of Dental Surgery .... 468

Mississippi Valley Dental Association .... 469

Alumni Meeting of Ohio College of Dental Surgery .... 469

### EDITORIAL, ETC.

Dr. E. Cutter .... 470

### BIBLIOGRAPHICAL.

Mechanical Dentistry. By Charles Hunter, England .... 472

The Advantages and Accidents of Artificial Anæsthesia. By Laurence Turnbull, M. D. 473

Facial Neuralgia. By J. Martin Kershaw, M. D. .... 473

Virginia Medical Monthly .... 474

Elements of Dental Materia Medica and Therapeutics— By James Stocken, L. D. S., R. C. S. .... 475

On the Treatment of Placentia Prævia. By Jos. T. Johnson, M. D. .... 475

Treatise on Dental Caries. By Dr. E. Magitot .... 475

### MONTHLY SUMMARY.

Imbedding of a Piece of Tooth in the Tongue .... 476
Sugar .... 476
Anæsthetic for Children .... 477
Effect of Diet on Liquor Drinking .... 477
Camphor as a Hypnotic .... 478
Paper Teeth .... 478
Decision in a Vulcanite Suit .... 479
Borax as an Antiseptic .... 479
Neuralgic Toothache .... 480
Chloral as a Revulsive .... 480
Cuprum Ammoniatum in Neuralgia .... 480
Convulsions Caused by a Hair .... 480

